# Nationwide incidence and treatment pattern of retinopathy of prematurity in South Korea using the 2007–2018 national health insurance claims data

**DOI:** 10.1038/s41598-021-80989-z

**Published:** 2021-01-14

**Authors:** Eun Hee Hong, Yong Un Shin, Gi Hwan Bae, Young Jin Choi, Seong Joon Ahn, Lucia Sobrin, Rimkyung Hong, Inah Kim, Heeyoon Cho

**Affiliations:** 1 Department of Ophthalmology, Hanyang University College of Medicine, Seoul, Korea; 2 Department of Occupational and Environment Medicine, Hanyang University College of Medicine, Seoul, Korea; 3 Department of Pediatrics, Hanyang University College of Medicine, Seoul, Korea; 4 Department of Ophthalmology, Massachusetts Eye and Ear Infirmary, Harvard Medical School, Boston, MA USA

**Keywords:** Eye diseases, Epidemiology, Paediatric research

## Abstract

The aim of this study is to investigate the nationwide incidence and treatment pattern of retinopathy of prematurity (ROP) in South Korea. Using the population-based National Health Insurance database (2007–2018), the nationwide incidence of ROP among premature infants with a gestational age (GA) < 37 weeks (GA < 28 weeks, GA28; 28 weeks ≤ GA < 37 weeks; GA28-37) and the percentage of ROP infants who underwent treatment [surgery (vitrectomy, encircling/buckling); retinal ablation (laser photocoagulation, cryotherapy)] were evaluated. We identified 141,964 premature infants, 42,300 of whom had ROP, with a nationwide incidence of 29.8%. The incidence of ROP in GA28 group was 4.3 times higher than in GA28-37 group (63.6% [2240/3522] vs 28.9% [40,060/138,442], *p* < 0.001). As for the 12-year trends, the incidence of ROP decreased from 39.5% (3308/8366) in 2007 to 23.5% (2943/12,539) in 2018. 3.0% of ROP infants underwent treatment (25.0% in GA28; 1.7% in GA28-37); 0.2% (84/42,300) and 2.9% (1214/42,300) underwent surgery and retinal ablation, respectively. The overall percentage of ROP infants who underwent treatment has decreased from 4.7% in 2007 to 1.8% in 2018. This first Korean nationwide epidemiological study of ROP revealed a decreased incidence of ROP and a decreased percentage of ROP infants undergoing conventional treatment during a 12-year period.

## Introduction

Retinopathy of prematurity (ROP) is a vision-threatening disease^1,2^ which affects the blood vessels of the developing retina and occurs primarily in premature infants^3^. It is the most widely recognized cause of visual impairment after preterm birth^2^. A meta‐analysis of 13 population‐based studies reported an annual incidence of 20,000 infants blind from ROP^2^. The proportion of blindness in children attributable to ROP ranges from 10 to 37.4% worldwide^4,5^.

There have been population-based studies of ROP in several countries, with different definitions used and different outcomes reported^6–10^. In the United States, the incidence of ROP among newborns with length of hospital stay (LOS) of more than 28 days was 12.8–19.9% in the studies using the publicly available pediatric inpatient care databases^6,7^. A recent study in Taiwan reported an ROP incidence of 36.6% among premature infants with LOS of more than 28 days using the National Health Insurance Research Database (NHIRD)^8^. In a Swedish national registry data, ROP was found in 31.9% of infants with a gestational age (GA) of < 31 weeks^9^, whereas in England, a dataset derived from the National Health Service (NHS) database revealed that 12.6% of babies with birth weight (BW) less than 1500 g had ROP in 2011^10^. In a Turkish neonatal intensive care units (NICUs) network, any stage of ROP was seen in 27–30% of infants with BW ≤ 1500 g, GA ≤ 32 weeks or with an unstable clinical course^11,12^. In South Korea, there was no population-based epidemiological study until a survey of the Korean Neonatal Network (KNN) database, a national registry for very-low-birth-weight infants (VLBWI; less than 1500 g BW), reported the total incidence of ROP to be 34.1% among VLBWIs^13^. However, the need for studies of the nationwide epidemiology of ROP in South Korea has remained as the KNN data covered about 70% of the overall admissions of VLBWIs born in the nation at that time^14^.

Treatment of ROP, to date, is largely by retinal ablation including cryotherapy or laser photocoagulation^15^, and also includes surgical procedures including vitrectomy and scleral buckling/encircling^16–19^. Recently, the efficacy of intravitreal anti-vascular endothelial growth factor (VEGF) in ROP has been demonstrated in the form of mono or combination therapy^20,21^. The safety of the anti-VEGF drugs is not yet completely established in infants though there has been widespread use of anti-VEGF injections since the 2010s, which has influenced treatment patterns of ROP worldwide^21,22^. There have been several studies regarding to the impact of anti-VEGF treatment in ROP in South Korea^23,24^, however, none demonstrated the treatment trend in ROP in this anti-VEGF era.

The National Health Insurance (NHI) database in South Korea allows researchers to obtain population-based, epidemiological data. It includes information on the total population and provides big data, including information on disease occurrence and treatment in the entire population. Although the use of intravitreal anti-VEGF in ROP was not covered by NHI service and was not registered to NHI database during the study period, the use of conventional treatment could be analyzed. Therefore, in this current study, we used the NHI database to investigate (1) the nationwide incidence of ROP among preterm infants, and (2) the percentage of patients with ROP who have undergone treatment (retinal ablation or surgery) from 2007 to 2018 in South Korea.

## Methods

This study was approved by the Institutional Review Board (IRB) of Hanyang University Guri Hospital, Gyunggi-do, South Korea. The requirement for written informed consent was waived because of the retrospective design (IRB no. 2018-04-001). The research was conducted according to the tenets of the Declaration of Helsinki.

### Database

We used health claims data recorded between 2007 and 2018 in the Korean National Health Insurance Service (KNHIS) database. In South Korea, the health security system provides healthcare coverage to all citizens, which has two components: National Health Insurance (NHI) and Medical Aid. > 97% of the population is covered by NHI, which is the single national insurance provider in South Korea and a compulsory health insurance, and the remaining 3% of the population is covered by the Medical Aid program, a public assistance program providing healthcare for the poor^25^. The KNHIS database covers all NHI beneficiaries and Medical Aid recipients in South Korea, and includes data regarding diagnoses, procedures, prescription records, medical treatment records, sociodemographic characteristics, and direct medical costs for claims made. Patients in the KNHIS database are identified by a unique identification number (Korean Resident Registration Number) assigned to each Korean resident at birth; therefore, health care records can be used without any duplications or omissions. In addition to the KNHIS database, annual data for the number of total newborns during the study period was downloaded from the Korean Statistical Information Service (KOSIS) website (http://kosis.kr/eng/).

### Cohort and case definition

In this study, cases were identified by the International Classification of Diseases, 10th edition (ICD-10). The KNHIS database manages claims using the Korean Classification of Disease (KCD), sixth edition, a modified version of the ICD-10, adapted for the Korean healthcare system. Premature infants were identified using the diagnostic code of “Extreme immaturity (P07.2, GA < 28 weeks)” or “Other preterm infants (P07.3, 28 weeks ≤ GA < 37 weeks)”, referred to as the GA28 group and the GA28-37 group, respectively.

Cases with congenital anomalies or perinatal injury which might affect the normal development were excluded as follows: encephaly (Q00.0–2), encephalocele (Q01.0–2,8), microcephaly (Q02), congenital hydrocephalus (Q03), other congenital malformations of brain (Q04.0–9), other disturbances of cerebral status of newborn (P91), disorders of muscle tone of newborns (P94), birth trauma (P10–15), intracranial non-traumatic hemorrhage of fetus and newborn (P52), intrauterine hypoxia (P20), and birth asphyxia (P21).

The definition of ROP was based on the diagnostic code of ROP (H351), within 180 days of the diagnosis of premature infant (P07.2–3).

### Incidence and treatment pattern in patients with ROP

The incidence of ROP among premature infants in this nationwide population was calculated by dividing the number of patients who were diagnosed with ROP by the total number of premature infants. The annual cumulative incidence (%) was calculated, starting on 1 January of each year between 2007 and 2018. The incidence rates of ROP by sex, GA (GA28 vs GA28-37), and year were also calculated.

Patients who underwent treatment were identified using procedure codes for pars plana vitrectomy (S5121-2), retinal detachment surgery (S5130), retinal photocoagulation (S5160), or cryopexy (S5140). To exclude treatment for other diseases, we included only cases with procedure codes of S5121-2, S5130, S5160, and S5140 within a year after the diagnosis of ROP. ROP infants who underwent treatment, referred to as the total treatment group, were defined as cases with at least one of all these procedure codes. ROP cases with (1) at least one of the procedure codes for pars plana vitrectomy or retinal detachment surgery (scleral buckling or encircling) were categorized as surgery subgroup; (2) at least one of the procedure codes of retinal photocoagulation or cryopexy were categorized as retinal ablation subgroup. The percentage of ROP infants who underwent each treatment subgroup were compared within each GA group (GA28 and GA-28-37 groups).

### Statistical analysis

The cumulative incidence (%), with 95% confidence intervals (CIs), were estimated using Poisson distribution. Odds ratios (ORs) with CIs were estimated using logistic regression analysis adjusted for sex, age, year of diagnosis, income level (grouped based on income quintiles), and area of residence (metropolitan cities and others). All two-sided p-values < 0.05 were considered statistically significant. All analyses were conducted using SAS version 9.4 (SAS Inc, Cary, NC).

## Results

### Patient demographics

**Figure 1:**
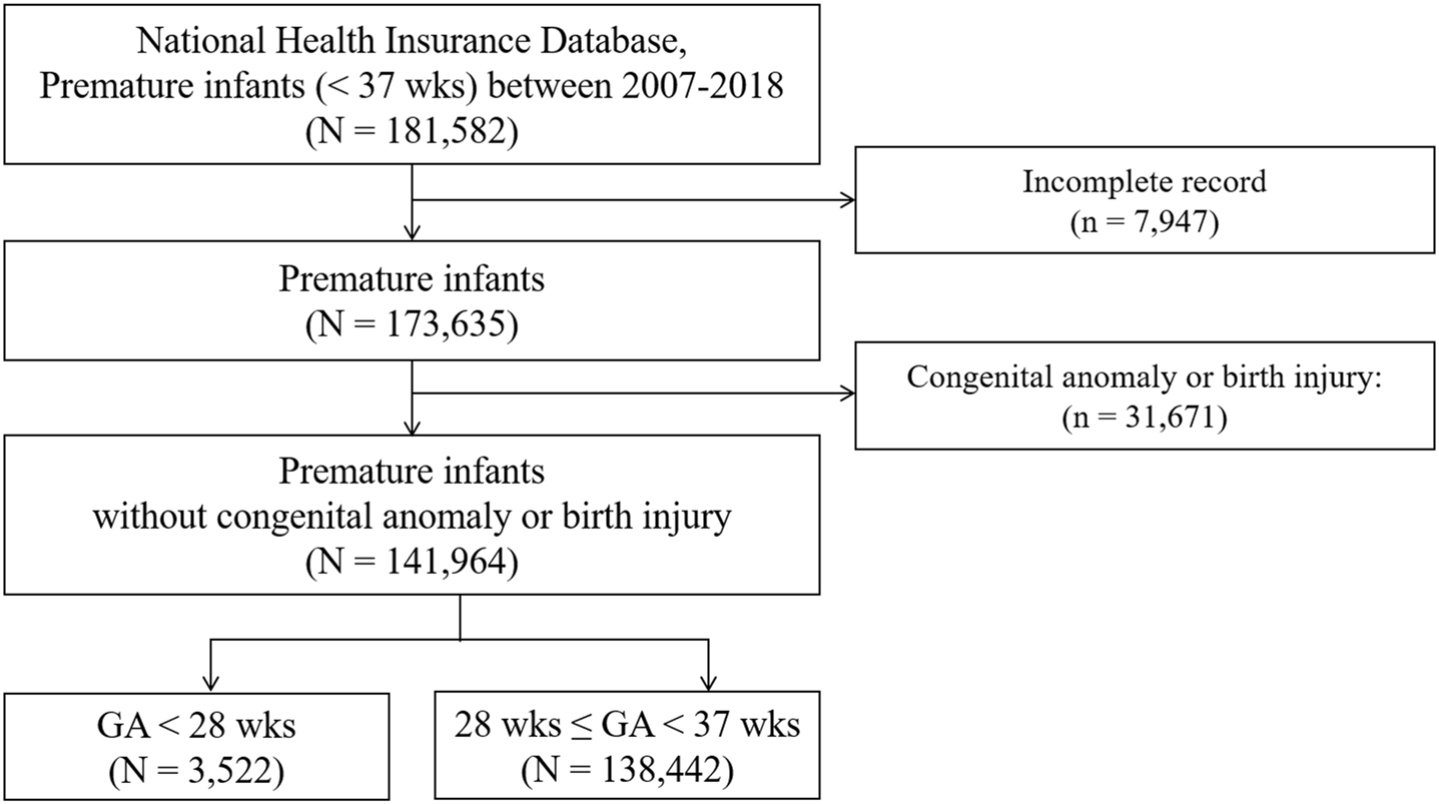
Flow chart illustrating the process of creating the cohort. Premature infants with a gestational age (GA) < 37 weeks who were born between 2007 and 2018 were enrolled. *GA* gestational age, *ROP* retinopathy of prematurity.

In total, 181,582 premature infants with a GA < 37 weeks were identified in South Korea during the 12-year period analyzed (2007 through 2018). Among them, 7947 cases with incomplete records and 31,671 cases with congenital anomaly or perinatal injury were excluded. The remaining 141,964 premature infants comprised the present study group. Figure 1 shows the flowchart of the enrollment of the study subjects. The overall and annual number of total newborns and premature infants is presented in Supplementary Table S1 online. The number of total newborns has decreased from 496,822 in 2007 to 326,822 in 2018, whereas the number of premature infants has increased from 8366 in 2007 to 12,539 in 2018.

### Incidence of ROP among premature infants

**Figure 2:**
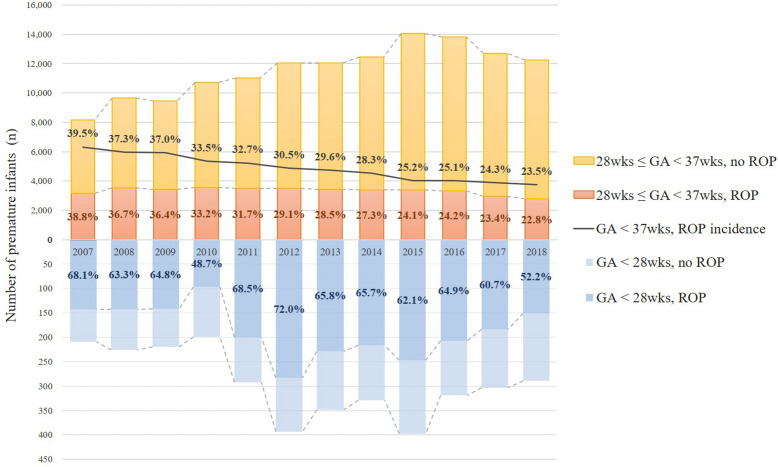
Twelve-year trend of incidence of retinopathy of prematurity (ROP) among premature infants according to gestational age (GA) groups. The stacked bar graph above the horizontal axis indicates the annual number of premature infants of 28 weeks ≤ GA < 37 weeks, with and without ROP, and incidence of ROP (%). The stacked bar graph below the horizontal axis indicates the annual number of premature infants of GA < 28 weeks, with and without ROP, and incidence of ROP (%). The line graph indicates the annual incidence of ROP among premature infants with a GA < 37 weeks. *GA* gestational age, *ROP* retinopathy of prematurity.

ROP was found in 42,300 newborns during the study period (total incidence, 29.8%). Table 1 shows the annual and overall cumulative incidences of ROP in premature infants according to age group and sex. The cumulative incidence of ROP among premature infants has decreased from 39.5% in 2007 to 23.5% in 2018; from 68.1 to 52.2% in the GA 28 group, from 38.8 to 22.8% in the GA28-37 group (Fig. 2). The overall incidence of ROP during the 12-year study period was 4.29 times higher in the GA28 group than in the GA28-37 group (adjusted OR 4.29, *p* < 0.001) and 0.97 times lower in males than females (adjusted OR 0.97, *p* < 0.001; Table 2).

**Table 1:** The cumulative incidence of retinopathy of prematurity in premature infants from 2007 to 2018 according to gestational age and sex.

Year	Total			Male			Female		
	Person-year	ROP (n)	Cumulative incidence (%)	Person-year	ROP (n)	Cumulative incidence (%)	Person-year	ROP (n)	Cumulative incidence (%)
**Total (GA < 37 weeks)**									
2007	8366	3308	39.5	4520	1799	39.8	3846	1509	39.2
2008	9890	3687	37.3	5456	1986	36.4	4434	1701	38.4
2009	9667	3579	37.0	5167	1922	37.2	4500	1657	36.8
2010	10,926	3656	33.5	5901	1958	33.2	5025	1698	33.8
2011	11,330	3702	32.7	6240	2005	32.1	5090	1697	33.3
2012	12,451	3794	30.5	6770	2027	29.9	5681	1767	31.1
2013	12,414	3673	29.6	6830	2021	29.6	5584	1652	29.6
2014	12,790	3623	28.3	6949	1949	28.0	5841	1674	28.7
2015	14,449	3635	25.2	7917	1970	24.9	6532	1665	25.5
2016	14,139	3546	25.1	7715	1927	25.0	6424	1619	25.2
2017	13,003	3154	24.3	7141	1722	24.1	5862	1432	24.4
2018	12,539	2943	23.5	6927	1619	23.4	5612	1324	23.6
Overall	141,964	42,300	29.8	77,533	22,905	29.5	64,431	19,395	30.1
**GA < 28 weeks**									
2007	207	141	68.1	92	61	66.3	115	80	69.6
2008	226	143	63.3	123	87	70.7	103	56	54.4
2009	219	142	64.8	110	71	64.5	109	71	65.1
2010	199	97	48.7	85	38	44.7	114	59	51.8
2011	292	200	68.5	131	82	62.6	161	118	73.3
2012	393	283	72.0	208	138	66.3	185	145	78.4
2013	348	229	65.8	171	115	67.3	177	114	64.4
2014	329	216	65.7	163	109	66.9	166	107	64.5
2015	398	247	62.1	216	130	60.2	182	117	64.3
2016	319	207	64.9	170	106	62.4	149	101	67.8
2017	303	184	60.7	153	93	60.8	150	91	60.7
2018	289	151	52.2	128	68	53.1	161	83	51.6
Overall	3522	2240	63.6	1750	1098	62.7	1772	1142	64.4
**28 weeks ≤ GA < 37 weeks**									
2007	8159	3167	38.8	4428	1738	39.3	3731	1429	38.3
2008	9664	3544	36.7	5333	1899	35.6	4331	1645	38.0
2009	9448	3437	36.4	5057	1851	36.6	4391	1586	36.1
2010	10,727	3559	33.2	5816	1920	33.0	4911	1639	33.4
2011	11,038	3502	31.7	6109	1923	31.5	4929	1579	32.0
2012	12,058	3511	29.1	6562	1889	28.8	5496	1622	29.5
2013	12,066	3444	28.5	6659	1906	28.6	5407	1538	28.4
2014	12,461	3407	27.3	6786	1840	27.1	5675	1567	27.6
2015	14,051	3388	24.1	7701	1840	23.9	6350	1548	24.4
2016	13,820	3339	24.2	7545	1821	24.1	6275	1518	24.2
2017	12,700	2970	23.4	6988	1629	23.3	5712	1341	23.5
2018	12,250	2792	22.8	6799	1551	22.8	5451	1241	22.8
Overall	138,442	40,060	28.9	75,783	21,807	28.8	62,659	18,253	29.1

*GA* gestational age, *ROP* retinopathy of prematurity.

**Table 2:** The odds ratios (OR) of incidence of retinopathy of prematurity according to gestational age and sex during the 12-year study period.

	Person-year	ROP (n)	Cumulative incidence (%)	Crude			Adjusted		
				OR	95% CI	P-value	OR	95% CI	P-value
GA < 28 weeks	3522	2240	63.6%	4.29	4.00–4.60	< .0001	4.29^a^	4.00–4.59	< .0001
28 weeks ≤ GA < 37 weeks	138,442	40,060	28.9%	Reference					
**Total (GA < 37 weeks)**									
Male	77,533	22,905	29.5%	0.97	0.95–1.00	0.022	0.97^b^	0.95–1.00	< .0001
Female	64,431	19,395	30.1%	Reference					
**GA < 28 weeks**									
Male	1750	1098	62.7%	0.93	0.81–1.07	0.293	0.93^b^	0.81–1.06	0.040
Female	1772	1142	64.4%	Reference					
**28 weeks ≤ GA < 37 weeks**									
Male	75,783	21,807	28.8%	0.98	0.96–1.01	0.147	0.98^b^	0.96–1.01	< .0001
Female	62,659	18,253	29.1%	Reference					

OR was estimated using logistic regression analysis.

*GA* gestational age, *ROP* retinopathy of prematurity.

^a^OR adjusted for sex, year of diagnosis, income level, area of residence.

^b^OR adjusted for year of diagnosis, income level, area of residence.

### Treatment of ROP

**Figure 3:**
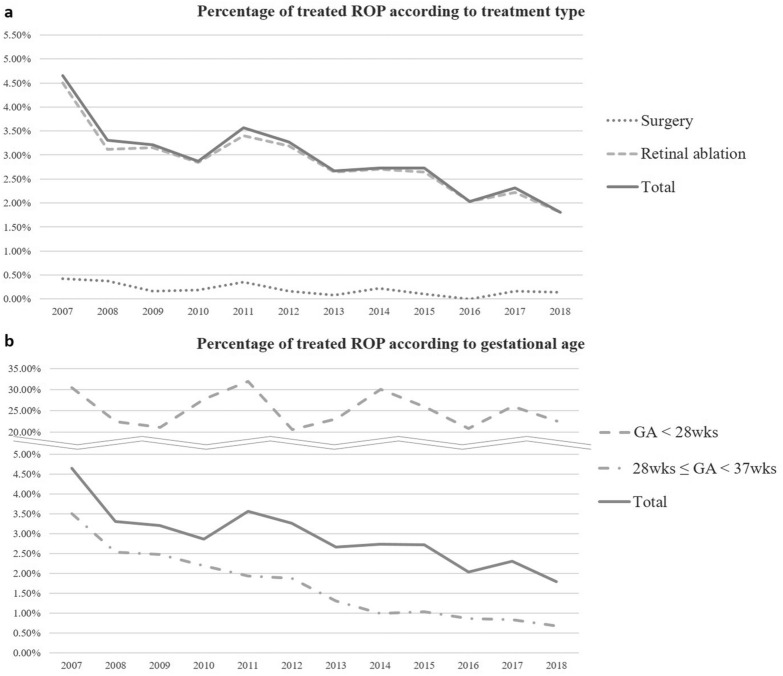
Twelve-year trend of treatment in retinopathy of prematurity (ROP) according to the treatment type and gestational age (GA) groups. The annual percentage of ROP infants who underwent treatment according to the treatment type (**A**) and the GA groups (**B**) are presented. GA = Gestational age; ROP = retinopathy of prematurity.

Among ROP patients, 3.0% (1246/42,300) underwent treatment during the 12-year of study period. Surgery and retinal ablation subgroups represented 0.2% (84/42,300) and 2.9% (1214/42,300) of the ROP infants, respectively. The total treatment group accounted for 25.0% (561/2240) of ROP in the GA28 group and 1.7% (685/40,060) in the GA28-37 group (Table 3). Figure 3 shows the 12-year trend in the treatment pattern for ROP. The overall percentage of ROP infants who underwent treatment decreased during study period from 4.7% in 2007 to 1.8% in 2018, with the sharpest decline between 2007 (4.7%) and 2008 (3.3%). The proportion of each treatment subgroup in total and for each gestational group also has decreased (Supplementary Table S2 online).

**Table 3:** The percentage of patients with retinopathy of prematurity who underwent treatment during the 12-year of study period according to gestational age and sex.

Treatment category	Total			Male			Female		
	ROP (n)	Treatment		ROP (n)	Treatment		ROP (n)	Treatment	
		(n)	(%)		(n)	(%)		(n)	(%)
**Total (GA < 37 weeks)**									
Surgery		84	0.2		41	0.2		43	0.2
Retinal ablation		1214	2.9		606	2.7		608	3.1
Total	42,300	1246	3.0	22,905	621	2.7	19,395	625	3.2
**GA < 28 weeks**									
Surgery		31	1.4		15	1.4		16	1.4
Retinal ablation		550	24.6		266	24.2		284	24.9
Total	2240	561	25.0	1098	271	24.7	1142	290	25.4
**28 weeks ≤ GA < 37 weeks**									
Surgery		53	0.1		26	0.1		27	0.2
Retinal ablation		664	1.7		340	1.6		324	1.8
Total	40,060	685	1.7	21,807	350	1.6	18,253	335	1.8

*GA* gestational age, *ROP* retinopathy of prematurity.

## Discussion

This first Korean nationwide study of ROP encompassing 12 years of premature infants in South Korea provides informative results regarding the incidence and treatment pattern for ROP among premature infants with a GA < 37 weeks. The national incidence of ROP has decreased in South Korea over a 12-year period with an overall incidence of 29.8%. The percentage of infants treated with retinal ablation or surgery for ROP has also decreased, with an overall percentage of 3.0%.

The national incidence of ROP of 29.8% in South Korea is hard to compare with other population-based studies in other countries because the definition of included premature infants in each study is not the same; GA was used in the current study whereas different definitions of GA, BW or LOS were used in other studies. A recent study in Taiwan, using the NHIRD which is similar to NHI database in South Korea, reported annual incidence of between 31 and 41% from 2002 to 2011 among all premature infants with LOS of more than 28 days, which might lead to underestimation of the number of premature infants^8^. The Turkish studies based on the NICUs network reported similar incidences of 30% and 27% in 2011–2013 and 2016–2017, respectively; between 2011–2013 and 2016–2017 though the included premature infants were only those with BW ≤ 1500 g, GA ≤ 32 weeks or with an unstable clinical course^11,12^. In European countries, an ROP incidence of 31.9% was found among infants with a GA of < 31 weeks between 2008 and 2015 in Sweden using national registry data^9^, and an annual incidence of 11–14% was reported among infants with BW < 1500 g between 2007 and 2011 (total incidence of 12.6% in 1990–2011) in England using the dataset from NHS^10^. In the United States, a study using a 20% representative sample of all US hospital discharges reported an ROP incidence of 15.6% (58,722/376,961) from 1997 to 2005 among newborns with LOS > 28 days^6^, and another study using the largest publicly available pediatric inpatient care database in the US reported incidences of 18.4% (9630/52,451) in 2009 and 19.9% (10,483/52,720) in 2012 among newborns with LOS > 28 days^7^. In South Korea, there have been several single-institution-based incidence studies. Cho and Koo reported an ROP rate of 21% (41/194) in infants with GA < 37 weeks or BW < 2000 g and who received supplemental oxygen therapy in 1991–1992^26^, and another study reported an ROP rate of 14.1% (96/666) among premature infants with GA < 37 weeks or BW < 2500 g in 1990–1992^27^. A more recent study showed an ROP incidence of 11.9% (24/201) in Korean infants with BW between 1500 and 2000 g or GA < 34 weeks in 2009–2013^28^. The first population-based epidemiological study used the KNN database and reported a total incidence of ROP of 34.1% among VLBWIs between 2013 and 2014^13^.

Several studies have reported an increase in ROP incidence. The Swedish study, with the most similar study period, reported an increase in incidence from 26.8% (187/698) in 2008 to 36.8% (283/2015) in 2015. They suggested that the continuous increase in the survival of the immature infants might contribute to the increasing incidence of ROP (and of the frequency of treatment)^9^. The increase of ROP incidence in the US studies from 14.7% (6201/42,178) in 2000 to 19.9% (10,483/52,720) in 2012 was explained by the widespread implementation of ROP screening guidelines after 2006, and recent advances in life-preserving technologies which have led to increased survival of premature infants, and also increased population of babies at risk for ROP^7^. Meanwhile, the Taiwanese study from 2002 to 2011 showed stationary incidence during the 10-year period, with a gradual decrease in the number of both premature infants and ROP patients^8^. In the present study, the incidence of ROP has decreased from 39.5% in 2007 to 23.5% in 2018, with a decrease in both the GA 28 (from 68.1 to 52.2%) and GA28-37 (38.8–22.8%) groups. The most likely explanation herein is the substantial increase in premature infants. It was noted that the number of newborns has decreased by 34.2% (from 496,822 to 326,822) whereas the number of premature infants enrolled in this study has markedly increased almost 1.5 times in 2018 compared to 2007 (149.9%, from 8366 to 12,539). As the analysis based on the NHI database has the limitation that cases registered with a proper diagnostic code could be enrolled, there should be a discrepancy between the number included in the present study and demographic-based statistical data. Moreover, since the current study was designed to enroll premature infants without congenital anomaly or birth injury, the real number of premature infants is estimated to be more than the number identified in this study. Nonetheless, the reduction in the annual number of newborns and the rapid increase in the annual number of premature infants before 37 weeks of GA, "the high-risk newborns”, themselves have been identified as a crucial health issue in recent decades in South Korea^29,30^. Although the number of any stage of ROP has only slightly decreased (3308–2943), the incidence of ROP among preterm infants has largely decreased by 40.5% (from 39.5 to 23.5%). The progressive advances in the guidelines for management of premature newborns to prevent the development of ROP may contributed to this decrease^15,31^, as the KNN in South Korea has reported an overall improvement in neonatal outcomes including ROP treatment from 2013 to 2016^32^.

The overall incidence of ROP among premature infants was 4.3 times higher in the GA28 group (63.6%) than in the GA28-37 group (28.9%). Low gestational age is a well-known major risk factor for ROP^15,33^. The incidence of ROP among infants with GA ≤ 28 weeks in Turkey was reported to 52.8% in 2011–2013 compared to 27.6% in infants with GA 29–32 weeks, and 62.9% in 2016–2017 compared to 19.4% in infants with GA 29–32 weeks^11,12^. The Postnatal Growth and Retinopathy of Prematurity (G-ROP) study in the US and Canada showed an ROP incidence of 74.7% (2369/3173) in 2006–2011. In our analysis, the overall incidence of ROP was slightly (0.97 times), but significantly, lower in males than females. This is consistent with the US studies^6,7^, but differs from other studies reporting no difference in sex^34^, or the male preference^35^. Further studies are needed to conclusively determine the relationship between sex and ROP.

The frequency of treatment among ROP infants in South Korea was 3.0%, with a decrease from 4.7% in 2007 to 1.8% in 2018. Although there have been several nationwide studies reporting the treatment frequency in ROP, the included treatment modalities were different (Table 4). In addition to these nationwide studies, in the Turkish studies based on the NICUs network, infants with BW ≤ 1500 g, GA ≤ 32 weeks or with an unstable clinical course had a treatment frequency of 17.1% (810/4729, laser photocoaguoation and vitreoretinal surgery) in 2011–2013 and 24.4% (414/1695, laser photocoaguoation, intravitreal bevacizumab, and vitreoretinal surgery) in 2016–2017^11,12^. The low treatment percentage in the present study could be mostly accounted for by the study population which includes more infants with large GA compared to other studies. Among ROP infants with GA < 28 weeks, the treatment percentage of 25.0% is comparable to other studies. Another important factor, which also explains the decreased trend of conventional treatment, is the emergence of anti-VEGF treatment. As anti-VEGF treatment for ROP has become widespread, it has replaced conventional treatment in many cases^12,36^. In South Korea, a study using KNN database between January 2013 and June 2014 examined the percentage of ROP infants who received conventional treatment (cryotherapy or laser photocoagulation and/or vitrectomy) and anti-VEGF treatment^13^. Among ROP in VLBWIs, 33.7% (231/686) underwent treatment: 63.6% (192) underwent conventional treatment only; 16.9% (84) underwent anti-VEGF treatment only; 19.5% (45) underwent both conventional and anti-VEGF treatment. The trend of using intravitreal anti-VEGF for ROP in South Korea has not been studied other than this KNN report though it has been reported to have been used in clinics since the late 2000s^23,37–39^. A recent retrospective study conducted in a Korean tertiary hospital reported the use of anti-VEGF for the treatment of ROP since 2006^37^. They included 314 eyes between January 2006 to December 2016 to compare the outcome of primary intravitreal anti-VEGF treatment and laser photocoagulation for ROP; among those, 161 eyes were treated with laser treatment primarily and 153 eyes were treated with intravitreal anti-VEGF injection primarily. There were also other reports in which the combination of laser photocoagulation and intravitreal anti-VEGF were used for ROP^23,38,39^, one of which reported the use of anti-VEGF since 2006^38^. Overall, this implies that the decrease in use of conventional treatments, especially retinal ablation, since 2007 shown in the present study may reflect the increase in use of anti-VEGF injection in this anti-VEGF era in South Korea. The overall lower treatment percentage throughout the study period may also have been partly attributed to the use of anti-VEGF injections that could not be included using the health claim database.

**Table 4:** Nationwide studies of treatment in retinopathy of prematurity including retinal ablation and surgery in the last 10 years.

	Sweden^9^	Taiwan^8^	United States^7^	England^10^	Present Study
Study period	2008–2015	2002–2011	2006, 2009, 2012	1990–2011	2007–2018
Population	GA < 31 weeks	Premature infants with LOS > 28 days	Premature infants with LOS > 28 days	Infants with BW < 1500 g	GA < 37 weeks
ROP (n)	1,829	4,096	27,481	NA	42,300
Treatment modality	NA	Cryotherapy, laser photocoagulation, intravitreal injection, scleral buckle, vitrectomy	Laser photocoagulation, scleral buckle, vitrectomy	Cryotherapy, laser photocoagulation	Cryotherapy, laser photocoagulation, scleral buckle, vitrectomy
Treatment	18.0% (329/1829)	6.5%	8.31%	13.3% in 1990; 11.8% in 2011	2.95% (GA < 28 weeks: 25.0%)
Trend	No significant change	Increased from 2.0% in 2002 to 9.3% in 2011	NA	Decreased until 2006, Increased from 2005	Decrease from 4.66% in 2007 to 1.80% in 2018

*GA* gestational age, *LOS* length of hospital stay, *ROP* retinopathy of prematurity.

The strength of the present study is that it was a large population-based study using a nationwide database, which allowed us to investigate national trends over a 12-year period. In addition, the possibility of missing data is expected to be very low for prematurity and ROP because all of premature infants need admission to hospitals. Therefore, our findings provide a valuable understanding of national epidemiology of ROP in South Korea. There were several limitations in the current study. First, the study subjects were identified by ICD-10 diagnostic and procedure codes. The precise definition of the study subjects is important, so we excluded cases with congenital anomalies or birth injuries to avoid overestimation. However, this could have led to an underestimation of the number of premature infants. Second, there are some limitations related to code registration that we figured out while conducting this analysis. The registered diagnostic codes were mostly integrated rather than sub-divided (i.e., P07.30 [28 weeks ≤ GA < 32 weeks] and P07.31 [32 weeks ≤ GA < 37 weeks] under the integrated code of P07.3 [28 weeks ≤ GA < 37 weeks, GA28-37 group]), and only a small portion of the subjects were registered using the sub-divided codes. Therefore, we could not divide the GA28-37 group into smaller GA groups. Additionally, as it is not compulsory for clinicians to register both, the diagnostic codes of GA and BW, it seemed that the diagnostic code of BW was likely to be missing once that of GA was registered. Among our study population, the number of those with diagnostic codes with low BW (P07.0 [“Extremely low birth weight”, BW < 1000 g] and P07.1 [“Other low birth weight”, 1000 g ≤ BW < 2500 g]) were much smaller compared to the total number of premature infants, so the incidence of ROP according to BW among our study population could not be analyzed in the present study. Third, detailed information regarding the prematurity and additional disease details such as the severity of ROP were not available from NHI database. Therefore, we defined subgroups of premature infants according to GA, but could not correlate this with BW or severity of ROP. Last, we investigated the treatment patterns for ROP in South Korea, but the use of anti-VEGF treatment was not provided by NHI database. As the popularity of anti-VEGF treatment in ROP is increasing, standardized guidelines are needed. In addition, a further nationwide study of treatment trends in ROP including anti-VEGF treatment would give further insights into adoption of this newer treatment.

In conclusion, this is the first nationwide incidence and treatment trend study of ROP in South Korea using a population-based database over 12 years. A decrease in the incidence of ROP among premature infants was found, and the incidence was 0.97 times lower in males than in females, and 4.29 times higher in the GA28 group than in the GA28-37 group. The percentage of ROP infants who underwent conventional treatment also showed a decreasing trend during the study period. The decrease in conventional treatment may suggest the increasing use of anti-VEGF treatment, and the impact of anti-VEGF treatment should be investigated in an additional study. Finally, the findings of the present study may throw light on our understanding of the epidemiology of ROP in South Korea and help establish the national strategies in managing premature infants and ROP.

## Supplementary Information

Supplementary Information

## Data Availability

The data that support the findings of this study are available from the corresponding author upon reasonable request.
