# Supplementary material for: Nationwide incidence and treatment pattern of retinopathy of prematurity in South Korea using the 2007–2018 national health insurance claims data
Source: Sci Rep. 2021 Jan 14;11:1451. doi: 10.1038/s41598-021-80989-z (PMC7809441; doi:10.1038/s41598-021-80989-z)
Supplement: Supplementary file 1 — Supplementary Information [file 41598_2021_80989_MOESM1_ESM.pdf]

Nationwide incidence and treatment pattern of retinopathy of prematurity in South Korea using the 2007-2018 national health insurance claims data

Eun Hee Hong[1,+], Yong Un Shin[1,+], Gi Hwan Bae[2], Young Jin Choi[3], Seong Joon Ahn[1], Lucia Sobrin[4], Rimkyung Hong[1], Inah Kim[2,*], Heeyoon Cho[1,*]

[1]Department of Ophthalmology, Hanyang University College of Medicine, Seoul, Korea

[2]Department of Occupational and Environment Medicine, Hanyang University College of Medicine, Seoul, Korea

[3]Department of Pediatrics, Hanyang University College of Medicine, Seoul, Korea

[4]Department of Ophthalmology, Massachusetts Eye and Ear Infirmary, Harvard Medical School, Boston, MA, USA

*Corresponding Authors: Heeyoon Cho, MD, PhD, hycho@hanyang.ac.kr

*Co-corresponding Author: Inah Kim, MD, MPH, PhD, inahkim@hanyang.ac.kr

[+]These authors (Eun Hee Hong and Yong Un Shin) contributed equally to this work.

Supplementary Table S1. Annual number of total newborns and premature infants identified in this study during the study period (2007 through 2018).

	Total		Male		Female	
	**Total newborns**	**Premature infants (GA < 37 wks)**	**Total newborns**	**Premature infants (GA < 37 wks)**	**Total newborns**	**Premature infants (GA < 37 wks)**
**Year**						
**2007**	496,822	8,366	255,872	4,520	240,950	3,846
**2008**	465,892	9,890	240,119	5,456	225,773	4,434
**2009**	444,849	9,667	229,351	5,167	215,498	4,500
**2010**	470,171	10,926	242,901	5,901	227,270	5,025
**2011**	471,265	11,330	242,121	6,240	229,144	5,090
**2012**	484,550	12,451	248,958	6,770	235,592	5,681
**2013**	436,455	12,414	223,883	6,830	212,572	5,584
**2014**	435,435	12,790	223,356	6,949	212,079	5,841
**2015**	438,420	14,449	224,906	7,917	213,514	6,532
**2016**	406,243	14,139	208,064	7,715	198,179	6,424
**2017**	357,771	13,003	184,308	7,141	173,463	5,862
**2018**	326,822	12,539	167,686	6,927	159,136	5,612
**Overall**	5,234,695	141,964	2,691,525	77,533	2,543,170	64,431

GA, Gestational age

Supplementary Table S2. The annual percentage of ROP infants who underwent treatment according to the treatment type and the GA groups.

	Overall (GA < 37wks)				GA < 28wks				28wks ≤ GA < 37wks			
	**ROP (n)**	**Surgery (n, %)**	**Retinal ablation (n, %)**	**Total (n, %)**	**ROP (n)**	**Surgery (n, %)**	**Retinal ablation (n, %)**	**Total (n, %)**	**ROP (n)**	**Surgery (n, %)**	**Retinal ablation (n, %)**	**Total (n, %)**
**Year**												
**2007**	3308	14 (0.4%)	149 (4.5%)	154 (4.7%)	141	5 (3.6%)	40 (28.4%)	43 (30.5%)	3167	9 (0.3%)	109 (3.4%)	111 (3.5%)
**2008**	3687	14 (0.4%)	115 (3.1%)	122 (3.3%)	143	2 (1.4%)	32 (22.4%)	32 (22.4%)	3544	12 (0.3%)	83 (2.3%)	90 (2.5%)
**2009**	3579	6 (0.2%)	113 (3.2%)	115 (3.2%)	142	1 (0.7%)	29 (20.4%)	30 (21.1%)	3437	5 (0.2%)	84 (2.4%)	85 (2.5%)
**2010**	3656	7 (0.2%)	104 (2.8%)	105 (2.9%)	97	0 (0.0%)	27 (27.8%)	27 (27.8%)	3559	7 (0.2%)	77 (2.2%)	78 (2.2%)
**2011**	3702	13 (0.4%)	126 (3.4%)	132 (3.6%)	200	9 (4.5%)	59 (29.5%)	64 (32.0%)	3502	4 (0.1%)	67 (1.9%)	68 (1.9%)
**2012**	3794	6 (0.2%)	121 (3.2%)	124 (3.3%)	283	4 (1.4%)	57 (20.1%)	58 (20.5%)	3511	2 (0.1%)	64 (1.9%)	66 (1.9%)
**2013**	3673	3 (0.1%)	97 (2.6%)	98 (2.7%)	229	0 (0.0%)	53 (23.1%)	53 (23.1%)	3444	3 (0.1%)	44 (1.3%)	45 (1.3%)
**2014**	3623	8 (0.2%)	98 (2.7%)	99 (2.7%)	216	5 (2.3%)	65 (30.1%)	65 (30.1%)	3407	3 (0.1%)	33 (1.0%)	34 (1.0%)
**2015**	3635	4 (0.1%)	96 (2.6%)	99 (2.7%)	247	1 (0.4%)	64 (25.9%)	64 (25.9%)	3388	3 (0.1%)	32 (0.9%)	35 (1.0%)
**2016**	3546	0 (0.0%)	72 (2.0%)	72 (2.0%)	207	0 (0.0%)	43 (20.8%)	43 (20.8%)	3339	0 (0.0%)	29 (0.9%)	29 (0.9%)
**2017**	3154	5 (0.2%)	70 (2.2%)	73 (2.3%)	184	2 (1.1%)	47 (25.5%)	48 (26.1%)	2970	3 (0.1%)	23 (0.8%)	25 (0.8%)
**2018**	2943	4 (0.1%)	53 (1.8%)	53 (1.8%)	151	2 (1.3%)	34 (22.5%)	34 (22.5%)	2792	2 (0.1%)	19 (0.7%)	19 (0.7%)
**Overall**	42300	**84 (0.2%)**	**1214 (2.9%)**	**1246 (3.0%)**	2240	**31 (1.4%)**	**550 (24.6%)**	**561 (25.0%)**	40060	**53 (0.1%)**	**664 (1.7%)**	**685 (1.7%)**

GA, Gestational age; ROP, retinopathy of prematurity
